# Structure, Thermal, and Mechanical Behavior of the Polysulfone Solution Impregnated Unidirectional Carbon Fiber Yarns

**DOI:** 10.3390/polym15234601

**Published:** 2023-12-02

**Authors:** Dilyus I. Chukov, Victor V. Tcherdyntsev, Andrey A. Stepashkin, Mikhail Y. Zadorozhnyy

**Affiliations:** Laboratory of Functional Polymer Materials, National University of Science and Technology “MISIS”, Leninskii Prosp. 4, 119049 Moscow, Russia; dil_chukov@mail.ru (D.I.C.); a.stepashkin@misis.ru (A.A.S.); priboy38@mail.ru (M.Y.Z.)

**Keywords:** polysulfone, carbon fibers, solution impregnation, residual solvent, porosity, mechanical properties, thermal behavior

## Abstract

The paper is devoted to the study of thermal and mechanical behavior and structural features of the polysulfone solution impregnated unidirectional carbon fiber yarns depending on fabrication conditions and appearance for optimum production method of the composites. The effect of producing conditions, such as polysulfone solution concentration, drying and post-heating temperatures, and the residual solvent content on the structure, mechanical, and thermal properties of the carbon fiber-reinforced composites was studied. The polysulfone solution impregnated carbon fiber yarns show relatively high mechanical properties, realizing up to 80% of the carbon fibers’ tensile strength, which can be attributed to good wettability and uniform polymer matrix distribution throughout the entire volume of the composites. It was found that the composites impregnated with 40 wt.% of the polysulfone solution showed lower porosity and higher mechanical properties. The results of a dynamic mechanical analysis indicate that residual solvent has a significant effect on the composites’ thermal behavior. The composites heated to 350 °C for a 30 min showed higher thermal stability compared to ones dried at 110 °C due to removal of residual solvent during heating. The impregnated carbon fiber yarns can be used for the further producing bulk unidirectional composites by compression molding and the proposed method can be easily transformed to continuous filament production, for example for further use in 3-D printing technology.

## 1. Introduction

Carbon fiber-reinforced polymer-matrix composites are the some of the most high-performance composites in terms of mechanical properties and strength-to-weight ratio. This makes them attractive to use in various applications such as in aerospace, transportation, watercrafts, windmill blades, and so on. The reinforcing fibers in the composites are mainly presented by three types including short fibers, continuous fibers, and woven fabrics [1,2,3,4]. The last two types are preferable to achieve high strength and stiffness of the resulting composites. As a matrix of the composites, thermoset and thermoplastic polymers can be used. Thermosets (epoxy, phenolic resins, etc.) are mainly liquid polymers at a room temperature and they can easily impregnate the carbon fiber bundle forming the prepreg sheet that is a flexible material prior to hardening [5,6,7]. However, thermoset resins need large curing times to form final composite parts because they undergo crosslinking reactions during heating. On the other hand, thermoplastic polymers can reversibly repeat the solid and the molten state at room temperature and at high temperature, respectively, without any chemical reactions. This means that the composites can be remelted and reshaped several times and have excellent recyclability, as well as molding can be carried out in a very short time. Thermoplastic polymer-based composites are attractive materials due to their functional properties: high thermal stability, crack resistance, impact toughness, and chemical resistance [1,8,9]. 

Among the most-widely used high performance polymer matrices for the CF reinforced composites, such as polyphenylene sulfide and polyetheretherketone, the polysulfones group are very attractive for composite production. Polysulfone (PSU) possesses an excellent balance of properties, including high temperature resistance (up to 200 °C), mechanical properties (tensile strength up to 70 MPa), dimensional stability, and low moisture absorption. The most important advantage of PSU compared to poly (ether ether ketone) (PEEK) is its significantly cheaper cost, that can be useful for producing wide consumption CF-reinforced composites. Another weak point of the PEEK, despite most of studies and practical uses of thermoplastic composites being focused on the PEEK matrix [10,11,12], is its glass transition temperature. It is well known that for the most thermoplastic polymers, a noticeable drop of mechanical properties occurs due to a loss of thermal stability at temperatures above the glass transition temperature. For example, Li et. al. [13] investigated the dynamic mechanical properties of CF/PEEK composites and showed that at a temperatures above 125 °C, PEEK changed from a glass state to a highly elastic state, resulting in a sharp decrease in the storage modulus (at about five times). At the same time, CF-reinforced composites based on polysulfones showed stable behavior of the storage modulus up to 160–180 °C [14,15]. Also, the fully amorphous structure of the PSU avoids the need for strict control of the cooling rate during composite production. Meanwhile, for the PEEK-based composites, crystallization kinetics of the polymer matrix can be an important factor influencing the composite performance properties [16,17].

As it is known, moisture absorption can affect the mechanical behavior of fiber-reinforced composites seriously, and this effect depends not only on the matrix polymer nature, but on various other factors also. For instance, in [18], the effect of water aging of polycarbonate reinforced with CF at different temperatures was studied. The moisture absorption value was varied from 0.3% for aging at ambient temperature to 0.8% for aging at 80 °C, where aging at ambient temperature had nearly no effect on the mechanical behavior, whereas aging at 80 °C resulted in a drastic fall in the flexural strength. Study of ramie/carbon fiber-reinforced polyethylene terephthalate glycol-based hybrid composites showed that while the moisture absorption was about 2.5% for all studied samples, the decrease in the flexural strength varied from 25 to 40%, depending on the lay-up sequence of fiber fabric layers [19]. Investigation of poly(ether-ketone-ketone) filled with CF showed that, in spite of the moisture absorption being only 0.25%, combined treatment of such composites with moisture, temperature, and UV radiation resulted in a decrease in storage modulus from 40 to 10 GPa [20]. For CF-reinforced composites based on polyamide 6, the moisture absorption magnitude was found to be up to 7%. That level of moisture absorption results in a drastic drop in the mechanical properties of composites [21,22,23]. In [23], the effect of the CF state on the behavior of consolidated 3D printed polyamide-6 based composites was studied. It was observed that while the moisture absorption in the case of short CF-containing composites was about 4.5%, the use of continuous CF allowed the reduction of moisture absorption to 1 %. It should be noted that in the case of composites filled with continuous CF, moisture absorption has nearly no effect on the mechanical behavior of the composites [23].

Various approaches have been used to reduce the moisture absorption of CF-reinforced composites. As it was reported in [24], the addition of polypropylene CF-reinforced composites based on polyamide 6 allowed a decrease of the moisture absorption from 7 to 5.5%, where no effect of the addition on the mechanical properties was revealed. In [25], the effect of carbon fiber surface treatment by polyurethane dispersion on the mechanical behavior of the polyamide 6 reinforced with CF was studied. It was observed that such treatment did not have an affect on the moisture absorption value; the decrease in the mechanical properties with water aging was higher for composites with untreated CF. It was concluded that such surface treatment of CF contributes effectively to the improved resistance of the CF/polyamide 6 interface to a hydrothermal aging environment [25]. In [26], the effect on the surface coating of CF-reinforced composites based on polyamide 6 with polydopamine was investigated. It was observed that such coating allowed a decrease in the moisture absorption from 6 to 3%, with simultaneous significant improvement of composite mechanical behavior.

As it was mentioned, the nature of the matrix polymer affects the resistance of composites to the aggressive environment strongly. In [27], the effect of aging of polysulfone, polybutylene, and polyamide 6/6 in hot chlorinated water on the mechanical behavior of polymers. Polysulfone performed the best of the three materials with no discernable change in properties over the duration of the experiments, whereas the polyamide 6/6 showed a significant decrease in mechanical properties when the material was immersed in water [27]. This is a result of polyamide hydrolytic degradation of structure, accompanied with a drastic drop in polyamide 6/6 molar mass [28]. In addition to the matrix polymer properties, the nature of fillers can influence the moisture absorption properties of composites. As it was shown in [29], the moisture absorption for epoxy-based composites reinforced with both glass and carbon fabrics was nearly the same, whereas for polyphenylene sulfide-based composites, replacing the reinforcers from glass to carbon fabrics resulted in the decreases in moisture absorption magnitude from 0.2 to 0.06%. Thus, it can be concluded that the combination of polysulfones as matrix polymers with continuous carbon fibers as reinforcers provides excellent resistance to the combined effect of temperature changes and moisture absorption, which was confirmed in several experimental investigations [30,31,32]. It should be noted that the melt viscosity of the most of the high performance thermoplastics is much higher, being two to three orders of magnitude higher than thermoset polymers (in the range of 10^2^–10^4^ Pa·s) [33]. During the impregnation process, viscosity of the polymer matrix influences the impregnability of fibers, which is critical to the composites’ mechanical properties. As for the high viscosity polymer, it is difficult to completely impregnate the fiber bundles, and it results in a large number of pores and polymer-free regions forming in the final composites, accompanied with their poor mechanical properties. This means that commonly used composite production methods such as resin transfer molding, injection molding, winding molding, pultrusion forming, etc., can be extremely complicated for the continuous fiber-reinforced thermoplastic polymer matrix composites. The development of efficient composite molding technology is necessary to reduce production costs and facilitate the successful application of composites on an industrial scale.

Except for direct polymer melting methods, such as pultrusion and co-extrusion [34,35], currently, there are two main technologies that can be used for continuous fiber-reinforced thermoplastic polymer matrix composite production. One of them is bringing the fiber as close as possible to the thermoplastic polymer matrix before the composite consolidation, which includes powder impregnation [36,37,38], commingled yarn fabrication [39,40], co-woven fabric producing [41,42], and film stacking method [43,44,45] technologies, to reduce void and improve the matrix distribution. The other is synthesizing the thermoplastic polymer matrix directly in the composite fabrication step, such as in situ polymerization, due to the easier process of impregnation of the fiber bundles with low viscosity polymer precursors [46,47,48]. In a powder impregnation method, the reinforcing fiber yarn is continuously pulled through polymer powder suspensions in various liquids using pins or rollers, to open up the fiber tow and allow the polymer powder to penetrate between the filaments. The commingled yarn technique minimizes the distance between fibers and polymer matrix to provide better impregnation via commingling of the reinforcing fibers and thermoplastic fibers in a single yarn. In the film stacking method, polymer films and reinforcing fabrics are used directly, without going through prepreg. They are alternately interlaced and then fused together under heat and pressure. 

Most of the above-mentioned methods require preprocessing of thermoplastic polymers into suspension, fibers, and films, but do not solve two main problems in fiber-reinforced thermoplastics, such as high polymer melt viscosity, preventing penetration and uniform distribution of the polymer between the fiber filaments, and low wettability of most of the reinforcing fibers. Moreover, they all have some restrictions and need careful control of the impregnation process. For example, the film stacking method may be applicable only in the case of a low viscosity of the polymer matrix. For the powder impregnation method, it is difficult to handle the constituent materials since the polymer powder can be easily dislodged from the reinforcing filaments. In the commingled yarn method, the reinforcing fibers are easily damaged during the commingling process [49]. The main focus of all above mentioned methods is how to make thermoplastic matrices completely impregnate the reinforcing fibers and provide uniform distribution of the fibers in the entire volume of the composite. However, they commonly result in poor wettability and insufficient polymer distribution along the fiber axis as well as in the volume of the impregnated yarns due to high polymer melt viscosity. For example, in the case of using a one-step PEEK powder, suspension impregnation of the core/shell structure of the composites can be realized, where the polymer forms a shell on the CF yarn and barely penetrates to the internal volume of the yarns, resulting in a porous structure due to the non-infiltration of resin [50].

The polymer solution impregnation method can be an effective method for producing thermoplastic based continuous fiber-reinforced composites, since there are no problems associated with high polymer viscosity and fiber wettability when using the polymer solution technology. In this case, the polymer can be effectively embedded into the internal volume of the reinforcing fiber bundles, providing uniform distribution throughout the entire volume of the composites. The main problem in this case may be related to effective removal of the solvent after the fibers have been impregnated. Earlier [15,51], we showed that the solution impregnation method allows significant improvement of the quality of the fiber-reinforced composites compared to the melt impregnation ones in terms of polymer distribution and mechanical properties. In the our previous paper [52], PSU solution impregnated unidirectional carbon fiber yarns were produced and their deformation behavior under various mechanical test conditions, namely, under various loading rates, was studied. However, our previous studies have not studied, in detail, the effect of the residual solvent in the composites and the method of its removal on the mechanical and thermal behavior, as well as on the structural features of the resulting composites. It should be noted that the residual solvent can have a significant impact on the composites’ performance properties, since, during its removal, a defective and porous structure can be formed. The residual solvent can have the greatest impact on the thermal properties of the material. By embedding in and distributing through the polymer matrix, the solvent can separate polymer chains and weaken the intermolecular interaction of polymers, resulting in an increase in the mobility of polymer chains and, consequently, a decrease in *Tg* [53]. It is well known that for the polymers, especially for the amorphous polymers such as PSU, *Tg* is a limiting factor of their practical use at elevated temperatures. So, the purpose of this paper is a study of the thermal and mechanical behavior and structural features of PSU solution impregnated unidirectional carbon fiber yarns depending on fabrication conditions, and obtain the optimum fabrication conditions of the composites. The impregnated carbon fiber yarns can be used for the further producing bulk unidirectional composites by compression molding, and the proposed method can be easily transformed to continuous filaments production. For example, for further use in a 3-D printing technology. 

## 2. Materials and Methods

The matrix material used in this work was polysulfone (PSU) Ultrason S 2010 (BASF, Ludwigshafen, Germany), with a glass transition temperature of 187 °C, a density of 1.24 g/cm^3^, a tensile strength of 75.0 MPa, and a Young’s modulus of 2.6 GPa. The carbon fibers used were Toray T700SC–12K (Toray Industries, Inc., Tokyo, Japan); the fibers properties summarized in the Table 1.

To produce the PSU impregnated unidirectional carbon fiber yarns, a polymer solution impregnation method was used. The composites preparation was performed in 3 steps: (1) dissolving of the PSU in n-methylpyrrolidone (NMP) (Eastchem, Changzhou, China); (2) impregnation of the CFs with the PSU solution; (3) drying the composites to remove NMP. To produce the PSU solution, the polymer pellets were dissolved in n-methylpyrrolidone in a 1 L glass flask using overhead stirrers, IKA EUROSTAR 40 digital (IKA-Werke GmbH & Co., Staufen, Germany), at a rotation speed of 200 rpm and at an ambient temperature. By varying PSU to NMP mass ratios, various concentrations of the solution were prepared (20, 30 and 40 wt.% of PSU). The maximum PSU concentration of 40 wt.% was chosen because at a higher polymer content, the viscosity became too high and a uniform impregnation is more complicated. In addition, noticeable CF damage was detected. On the other hand, at a PSU content lower than 20 wt.%, an insufficient amount of the polymer matrix forms, which results in poor mechanical properties of the resulting composites. 

**Figure 1:**
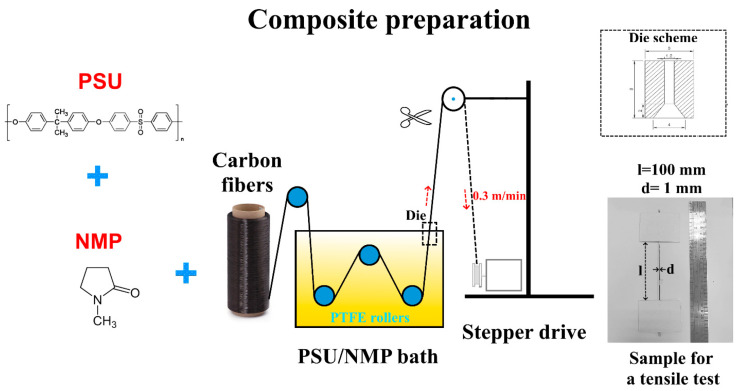
Scheme of the PSU impregnated unidirectional carbon fiber yarns preparation.

The impregnation process was carried out as described in Figure 1: a homemade PTFE bath with three rotatable PTFE guide rollers fixed in it was filled with a PSU solution of the desired concentration (20, 30, or 40 wt.%). Carbon fiber yarns were passed through the bath of the PSU solution. Flattening the carbon fibers onto PTFE rollers allowed PSU to penetrate into the bundles’ internal volume, resulting in a more uniform polymer distribution. At the top of the bath, a PTFE die with an internal diameter of 1.6 mm was fixed. Using a stepper drive, the carbon fibers were pulled through the die and excess polymer was removed at the die. The stepper drive’s rotation provided a linear speed of fiber passage through the bath equal to 0.3 m/min. When the length of the impregnated composites reached 1.5 m, the stepper drive stopped, and the impregnated yarns were wound on a metallic frame and dried at 115 °C for 4 h to remove solvent in a Binder FD-115 dryer (BINDER GmbH, Tuttlingen, Germany). Additionally, part of the dried composites was fixed on a metallic plate and held at 350 °C for 30 min in a furnace in an air atmosphere to additionally remove residual solvent. 

Each impregnated yarn was weighed on an analytical balance and its length was determined. The fiber mass fraction (*M_f_*) in the resulting composites was calculated on samples of 250–300 mm long using following equation:

$$M_{f}=\frac{m_{f}}{m_{c}}=\frac{\lambda_{m}*l_{f}}{m_{c}}*100\%$$

where *λ_m_* is the CF’s linear density, *l_f_* is the fibers length equal to the composite length, and *m_c_* is the composite mass.

All samples were marked according to the calculated fiber mass fraction. The round shaped impregnated yarns with an average diameter of 1 mm were glued into cardboard frames, as shown in Figure 1, for further tensile tests. Tensile tests of the produced unidirectional composites were performed according to the ISO 10618 standard [54] on a Zwick/Roell Z020 universal tensile testing machine equipped with a high-precision MultiXtens contact strain measurement system at a constant strain rate of 1.0%/min. The test samples were at least 250 mm in total length and had a 100 mm gauge length. For each type of composite, at least 15 specimens were tested. 

The composites’ density and porosity were determined according to ASTM D792 [55] and ASTM D2734-09 [56], respectively. Porosity was calculated as follow using theoretical (*ρ_t_*) and experimental (*ρ_exp_*) densities:

$$V_{p}=\frac{\rho_{t}{-\rho }_{exp}}{\rho_{t}}*100\%,$$

where the theoretical density was calculated as:

$$\rho_{t}=\frac{1}{M_{f}/\rho_{f}+M_{m}{/\rho }_{m}}$$

where *M_f_*, *M_m_*, *ρ_f_*, and *ρ_m_* are the mass fractions and densities of the fibers and matrix, respectively.

The fracture surfaces of the composites were studied using a TESCAN VEGA Compact (JSK TESCAN, Brno, Czech Republic, scanning electron microscope). For the SEM studies, all specimens were sputter coated with thin layer of carbon (10–15 nm) to improve the electrical conductivity of the samples.

The dynamic mechanical properties were characterized using a DMA Q800 (TA Instruments, New Castle, DE, USA) dynamic mechanical analyzer. The specimens for the DMA tests were approximately 45 mm long. The measurements were performed at a tension mode with constant test parameters of a 5 °C/min heating rate in the temperature range from 30 to 220 °C, a frequency of 1 Hz, and deformation of 0.1%.

## 3. Results and Discussion

Due to the high viscosity of the most commonly used thermoplastics, melt impregnation of continuous fibers and woven fabrics is complicated. It is clear that the viscosity of a polymer solution significantly lower than the melt viscosity. This allows better penetration of the polymer into the fiber bundles as well as providing higher wettability of the fibers, resulting in a more uniform fiber/matrix distribution throughout the entire volume of the prepared composites. In this study, we used various PSU concentrations in NMP (20, 30 and 40 wt.%) to investigate the effect of the polymer content on the structural, mechanical, and thermal properties of the impregnated unidirectional carbon fiber yarns. 

The use of different concentrations of polymer solution was associated not only with a variation of the carbon fibers to polysulfone ratio in the resulting composites, but also with the fact that it affected the impregnation process features since the viscosity of the different PSU solutions also differed noticeably. Additionally, in this study, we also used various post-processing methods to obtain two types of composite samples after polymer impregnation. To obtain the first series of composites, the PSU solution impregnated unidirectional carbon fiber yarns were simply dried for 4 h at a temperature of 110 °C, while the second series of the composites after drying were additionally held at the recommended processing temperature of the PSU [57] of 350 °C for 30 min. During the first post-processing (drying at 110 °C), we removed solvent from the samples to produce PSU/CF composites, while when heating up to processing temperature, we expected further consolidation of the composites, as well as more effective residual solvent removal. 

Table 2 summarizes fiber mass fractions and calculated values of theoretical densities of the composites depending on the PSU solution concentration and the post-processing method. It is obvious that the use of a higher concentration of the PSU solution resulted in a decrease in the carbon fiber content in the composites. A higher polymer content in the solution results in a higher polymer content in the composite, thereby reducing the fiber content. For example, the fiber content of the composites impregnated with a 20 wt.% PSU solution and dried at 110 °C was 71.8%; while when using a 30 wt.% PSU solution, the fiber content decreased to 62.8 wt.% and reached a minimum value of 57.7 wt.%. for the 40 wt.% PSU solution impregnated composites. The calculated values of the theoretical density associated with the polymer-to-fiber ratio in the composites were also reduced because the PSU density is lower than the CF density. It should be noted that the calculated values of the fiber content for composites after drying and subsequent heating up to 350 °C were 3–5% higher than for composites dried at 110 °C and were 74.5, 67.0, and 62.3 wt.% for composites impregnated with 20, 30, and 40 wt.% of the PSU solution. This is because when calculating the fiber content, the mass of the samples is considered, and the mass fraction of the matrix is equal to the sum of the masses of the polymer and the residual solvent. During heating to 350 °C, the mass of the fibers does not change, but the residual solvent is removed. As a result, the mass of the samples decreases and the calculated value of the fiber content increases. The difference between the calculated values of the fiber content for the composites after drying and subsequent heating shows the residual solvent content. This means that we can conclude that the produced composites contain 3–5% residual solvent after drying at 110 °C, which, as shown below, has a significant effect on the thermal behavior of the composites.

**Figure 2:**
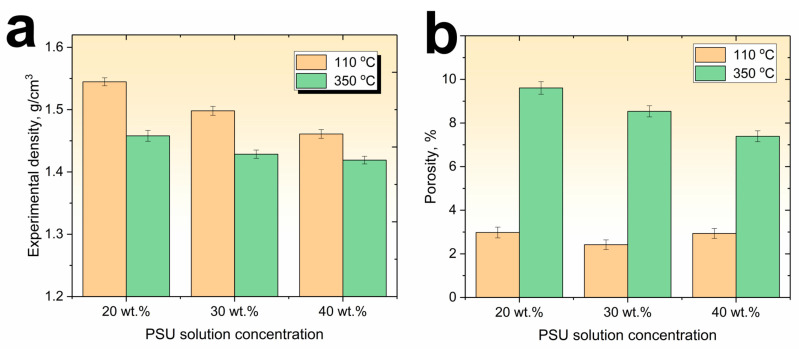
Experimental density (**a**) and porosity (**b**) values of the PSU impregnated unidirectional carbon fiber yarns.

Figure 2 shows the results of a study of the experimental density and the calculated porosity values of the PSU/CF composites. As expected, since the density of PSU is lower than the density of the carbon fibers, the density of the composites produced using a solution of a higher PSU concentration is lower for all the composites. So, if composites produced using a 20 wt.% PSU solution have an average experimental density of 1.54 g/cm^3^, the composites produced using 30 and 40 wt.% PSU solutions have average density values of 1.49 g/cm^3^ and 1.46 g/cm^3^, respectively.

**Figure 3:**
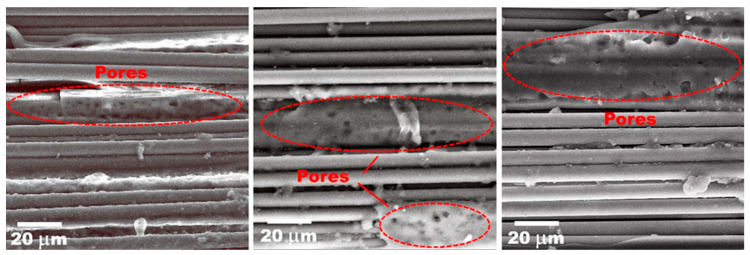
SEM images of the porous structure of the PSU impregnated unidirectional carbon fiber yarns.

It was found that the density of the composites after heating to 350 °C also decreased, and at a maximum concentration of the PSU solution of 40 wt.%, the density of the composites decreased from a value of 1.46 g/cm^3^ after drying at 110 °C to a value of 1.42 g/cm^3^ after heating to 350 °C. The reason for the observed decrease in the density values is pore formation processes as a result of boiling off the residual solvent during heating to polysulfone processing temperatures. Since it is known that the boiling point of n-methylpyrrolidone is 202 °C [58], when heated above these temperatures, the amount (3–5%) of residual n-methylpyrrolidone begins to boil away and is removed from the composite material, forming additional voids in the volume of the composite material. This is confirmed by an increase in the porosity of the composites after high temperature treatment (Figure 2b). So, for composites after drying at 110 °C the porosity values are in the range of 2.5–3% after post-heating to 350 °C, the porosity values increase up to 9.6, 8.5, and 7.3% for composites impregnated with 20, 30, and 40 wt.% polymer solutions, respectively. It can be seen that for the composites heated to 350 °C, an increase in the concentration of the PSU solution results in a decrease of porosity values because at a higher PSU solution concentration, a smaller volume of residual solvent remains in the composites. Therefore, when it boils away, fewer pores are formed. N-methylpyrrolidone forms a porous structure of the polymer while being removed from the composites volume (Figure 3), especially in the areas where a thick layer of polysulfone was formed between the carbon fiber bundles during the composite’s production. As it can be seen from the SEM images, the porosity of the produced composites is formed by two factors, including voids formed between individual carbon fiber filaments in the areas depleted of polymer, as well as pores formed directly in the volume of the polymer matrix when the solvent is removed. This means that using a higher polymer solution concentration reduces the factors that increase the porosity of the composites. 

**Figure 4:**
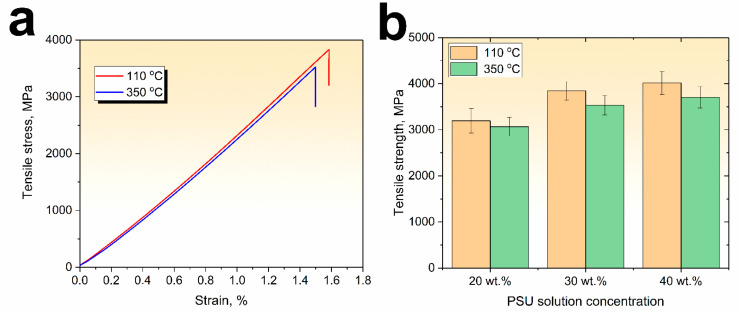
Typical stress–strain curves (**a**) after drying at 110 °C and 350 °C (for the 30 wt.% PSU) and tensile strength (**b**) of the various PSU solution impregnated unidirectional carbon fiber yarns.

To study the effect of the fiber to matrix ratio and thermal post-processing methods of the PSU/CF composites on their mechanical properties, tensile tests were performed. Figure 4a shows the tensile stress–strain curves of the 30 wt.% PSU solution impregnated unidirectional carbon fiber yarns fabricated under different conditions. The stress–strain curves of both composites (dried at 110 °C and heated at 350 °C) have a similar behavior: a nonlinear initial section up to a stress range of about 200 MPa, where the Young’s modulus increases from 180 to 220 GPa because of non-uniform loading of the CF filaments at the initial stage of the tensile tests. A further increase in the load and deformation up to the PSU yield point results in the polymer matrix beginning to flow in the direction of the applied load, allowing alignment of individual CF bundles achieving higher Young’s modulus values. It should be noted that the Young’s modulus of the composites is very close to the carbon fibers’ Young’s modulus. In addition, there was no noticeable difference between the Young’s modulus values of the composites impregnated with various PSU solution concentrations, which means good stress transfer ability of the polymer matrix regardless of the impregnation solution concentration used.

It was found that the PSU solution concentration affected on the tensile strength of the impregnated CF yarns. The tensile strength values of the PSU impregnated CF yarn depending on the polymer solution concentration and heating temperature are collected in Figure 4b. It can be seen that the tensile strength of composites impregnated with a 20 wt.% PSU solution and dried at 110 °C is about 3200 MPa, when using a 30 wt.% solution, the tensile strength increases to 3850 MPa, and it reaches a maximum value of 4020 MPa when using a maximum PSU solution concentration of 40 wt.%. An increase in the porosity of the composites after heating to 350 °C compared to the ones dried at 110 °C slightly decreases the mechanical properties. The tensile strength of the composites was 3070, 3530, and 3700 MPa for 20, 30, and 40 wt.% PSU solution impregnated carbon fiber yarns, respectively. The main number of the tested specimens had a brush-like failure. This type of failure is generally observed due to a poor fiber–matrix adhesion [59], which results in fiber/matrix debonding and splitting. In this failure mode, broken fibers are dispersed at any place along the specimen. In our previous studies [15,60], we have shown that commercially available carbon fibers mainly use epoxy-based sizing which is incompatible with PSU, neat CF show poor interfacial interaction, and additional surface modification may be needed to improve the fiber–matrix bonding. 

**Figure 5:**
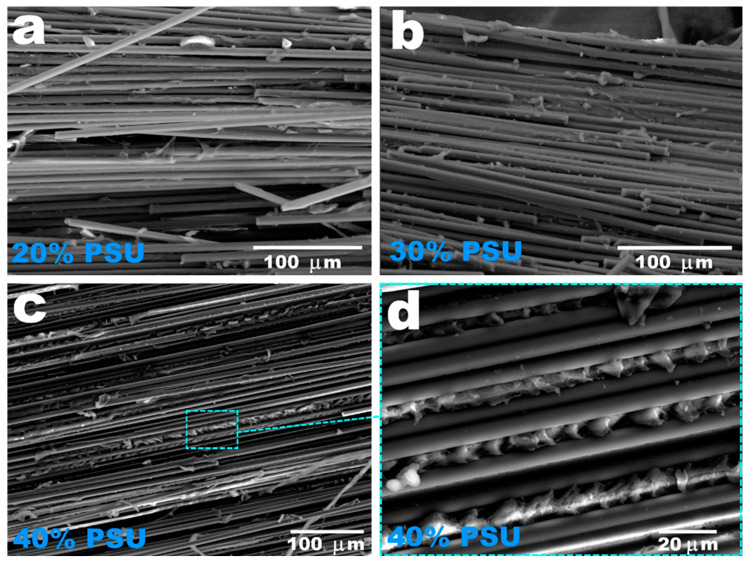
SEM images of the unidirectional carbon fiber yarns impregnated with 20 wt.% (**a**), 30 wt.% (**b**), and 40 wt.% (**c**,**d**) PSU solution.

SEM images confirmed the weak fiber–matrix interfacial interaction in the resulting composites (Figure 5). After tensile tests for all studied composites, the carbon fibers contained a small amount of polymer retained on the surface of the individual filaments. Analysis of the SEM images also showed that the lower the concentration of the PSU solution used, the less polymer matrix was formed in the internal volume of the composites. The fracture surface of the composites obtained by impregnation with a 20 wt.% PSU solution was highly depleted in the polymer. Because of that, they showed the lowest tensile strength values. When using a maximum concentration of a polymer solution of 40 wt.%, a fairly uniform distribution of the polymer throughout the entire volume of the composites was observed. In addition, as can be seen from Figure 5d, the polymer penetrated between individual filaments and polymer layers with a thickness of 7–10 μm were formed. 

Generally, a sufficient volume of polymer in the composite and its uniform distribution are the most important conditions for ensuring high mechanical properties of the resulting composites [61]. This is due to the fact that the matrix ensures the transfer of external load to the fibers and if areas depleted in polymer appear in the composite, it is usually impossible to achieve high mechanical properties, even if good adhesion of the polymer to the fibers is ensured. In this case, areas with an insufficient amount of matrix will be centers of initiation and propagation of cracks. As mentioned earlier, when using the polymer melt impregnation method, it is most often impossible to achieve homogeneity of the composites due to the high viscosity of thermoplastic polymers. But in this study, it was shown that despite the insufficient adhesion, the produced PSU solution impregnated CF yarns showed relatively high mechanical properties, realizing up to 80% of the carbon fibers tensile strength, which can be attributed to good wettability and uniform polymer matrix distribution throughout the entire volume of the composites. So, it can be concluded that solution impregnation can be an effective method for producing high performance carbon fiber-reinforced composites.

The static mechanical properties of composites only reflect the material properties macroscopically and cannot accurately indicate the changes in the internal matrix. Dynamic mechanical analysis (DMA) can be used to study the changes in composite materials under various conditions of alternating stress and temperature. Commonly, DMA analysis is an efficient way to evaluate the reinforcement, fiber surface modification, and pre- and post-treatment of composites by investigating the storage modulus, loss modulus, and loss factor (tan δ) of the composites. Moreover, as a result of the molecular motion of the chain segments, DMA can also provide precise information for the evaluation of relaxation/transition processes of the polymer matrix [13,62]. As in this study we used solution impregnation method, the effect of residual solvent on the thermal behavior of the composites is an important point.

**Figure 6:**
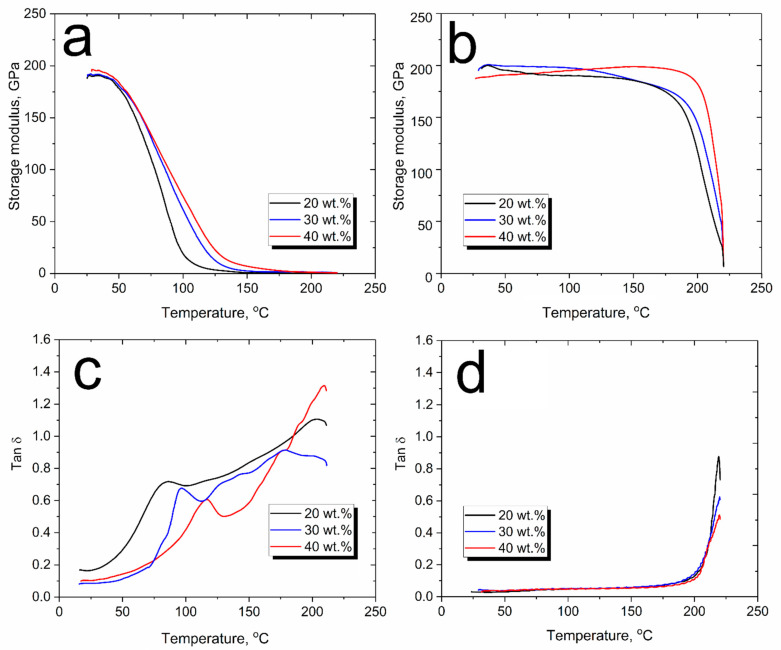
DMA results of the unidirectional carbon fiber yarns impregnated with 20 wt.%, 30 wt.%, and 40 wt.% PSU solution after drying at 110 °C (**a**,**c**) and heating to 350 °C (**b**,**d**).

The storage modulus E’ is an important parameter of stiffness and elasticity of composite materials, and the storage modulus of the PSU impregnated carbon fiber yarns varies with temperature, as shown in Figure 6. It is well known that the storage modulus of amorphous polymers decreases strongly in a temperature range around the glass transition temperature (*Tg*) [63]. As can be seen from Figure 6a, for composites that were dried from a solvent at a temperature of 110 °C, the storage modulus values are in the range of 190–200 GPa. It was found that there was a noticeable decrease in the storage modulus even at temperatures above 60 °C, and, with a further increase in temperature, the storage modulus dropped even more noticeably, reaching minimum values at temperatures above 150 °C. The temperatures of the start of the storage modulus’ drop only slightly shifted towards higher temperatures with an increase in the PSU solution concentration from 20 to 40 wt.%. While the end of the drop instorage modulus, which was determined as an intersection of two tangent lines from the storage modulus, occurred at different temperatures: 101, 124, and 133 °C for the 20, 30, and 40 wt.% PSU solution impregnated carbon fiber yarns, respectively. This phenomenon is related to the various residual solvent amounts in the composites produced using different PSU solutions. Usually, the drop in the storage modulus determines the maximum operating temperature of materials. Therefore, it can be concluded that the PSU impregnated carbon fibers yarns simply dried at 110 °C showed insufficient thermal behavior. It should be noted that the glass transition temperature of the neat PSU was 187 °C [59,62], and such a significant drop in the storage modulus is not typical for the PSU-based composites. In this study, the drop in the storage modulus was related to the presence of the residual solvent after drying at 110 °C, which increases the PSU molecular chains mobility. As it was mentioned earlier. By embedding in and distributing throughout the polymer matrix, the solvent can separate polymer chains and weaken the intermolecular interaction of the polymers, resulting in an increase in the mobility of the polymer chains and, consequently, a decrease in *Tg* [53]. It was found that for the CF impregnated with different PSU solution concentrations, a slightly different behavior of the storage modulus drop was observed. At a higher PSU solution concentration, the storage modulus drop caused temperatures shifts toward higher temperatures, which is related to the fact that at higher solution concentrations, the amount of residual solvent in the composite decreases, and, accordingly, the mobility of the PSU molecular chains decreases. 

The effect of the residual solvent on the thermal behavior of the material is further confirmed by studies of composites heated to 350 °C. Figure 6b shows a radically different behavior of the storage modulus dependence vs. temperature. As it can be seen, except for slight changes, the plateau of the storage modulus for all the composites can be observed up to a glass transition temperature of the PSU where a sharp decrease in the storage modulus occurs. The storage modulus drop’s temperatures were determined as 186, 194, and 203 °C, for the 20, 30, and 40 wt.% PSU solution impregnated carbon fiber yarns, respectively. The fact that the storage modulus drop’s temperatures were slightly higher than *Tg* of the neat PSU is because in the presence of reinforcements, the mobility of polymer chains is slightly restricted, which results in an increase in the thermal stability of highly filled composites. The stable behavior of the storage modulus of the composites heated at 350 °C is associated with the removal of the residual solvent, which increased the PSU chains’ mobility by weakening intermolecular interactions. Below the glass transition region (in the temperature range up to 180 °C), the solvent-free polymer’s chain movement is restricted due to the low mobility of the frozen and packed molecule arrangement. Thus, the storage modulus has a high and stable value in the glassy state. With the increase in temperature, the relative internal rotational motion of the molecular chains intensifies, resulting in a decrease in the load bearing capacity of the composites and, near the PSU glass transition temperature, the storage modulus drops and moves to the viscoelastic region of the material. It should be noted that in this case, the thermal behavior of the storage modulus of the solution impregnated composites is the same as for PSU melt impregnated composites produced in our earlier study [51]. 

Tan δ (loss factor) is defined as the ratio of the loss modulus to the storage modulus (tan δ = E″/E′); it is the ratio of the energy dissipated to the energy stored during a dynamic loading cycle. Usually, the maximum of the tan δ peak is related to the relaxation processes of the polymer matrix such as glass transition (α-transition), melting, or other. It can describe some features and changes of the internal structure of composite materials such as polymer chain mobility, glass transition, slip in the matrix/fiber interface, viscoplastic and thermoelastic damping, and so on. According to ASTM d 7028-07, the temperature at which a significant drop in the storage modulus begins is assigned as the glass transition temperature. The tan δ curve is a popular measurement point for the *Tg* and it is usually easier to isolate it than to determine the onset of the drop in the storage modulus [64]. At the same time, it should be noted that the *Tg* taken from a DMA curve is most often higher than a *Tg* measured using DSC [65].

Since the detected drop in the storage modulus of the composites simply dried at a temperature of 110 °C is associated with a noticeable decrease in the glass transition temperature of the PSU, analysis of the tan δ makes it possible to determine the influence of both the concentration of impregnating solutions and the heat treatment regimes of the composites on the thermal behavior of the resulting materials. Figure 6b,d show the tan δ vs. temperature plots for the composites impregnated with various concentrations of the PSU solution. A comparison of the tan δ values showed that for composites dried at a temperature of 110 °C, the glass transition temperatures of PSU obtained from tan δ peaks were 95.3, 106.2, and 126.4 °C, for composites obtained by impregnation with 20, 30, and 40 wt.% PSU solution, respectively. At the same time, for composites heated to 350 °C, the glass transition temperature determined from the tan δ peak was about 218 °C for the all the PSU solution concentrations. It can be concluded that the residual solvent in the composites dried at 110 °C dramatically decreases the PSU glass transition temperature.

The width of the tan δ peak can also be used to determine the structural features of a material. Generally, the width of the tan δ peak can indicate how homogeneous a system is; broader tan δ peaks indicate that the polymer has different chain lengths, and polymer systems with narrow peaks generally have a narrower distribution of chain types and molecular weights [66,67]. As it can be seen from Figure 6c,d, the composites dried at 110 °C showed significantly broader tan δ peaks compared to ones heated to 350 °C. In this case, the width of the tan δ peaks is not related to the PSU molecular chains’ length, but to the presence of different amounts of residual solvent in different regions of the polymer. Due to the nonuniform distribution of the residual solvent in different regions of the polymer, the mobility of the molecular chains differs, which broadens the tan δ peaks for composites dried at 110 °C. It can be seen that after heating to 350 °C, the composites are characterized by a rather narrow tan δ peak, which is caused by the removal residual solvent and the formation of a fairly homogeneous polymer structure. Comparison of the tan δ peak values also shows that the composites produced using higher polymer solution concentrations were characterized by lower values of the tan δ peak, which means the composites had a more elastic response. 

## 4. Conclusions

This paper describes the features of producing polysulfone impregnated unidirectional carbon fiber yarns. The effect of various post-processing methods of the impregnated yarns was studied. To obtain the first series of the composites, PSU solution impregnated unidirectional carbon fiber yarns were simply dried for 4 h at 110 °C, while the second series of the composites, after drying, were additionally heated up to a recommended processing temperature of the PSU of 350 °C for 30 min. 

The following conclusions can be drawn from this study:

- The composites after drying at 110 °C contained 3–5% residual solvent, while heating up to 350 °C allowed production of solvent-free composites.
- The composites simply dried at 110 °C showed insufficient thermal behavior, which was related to the presence of residual solvent after drying, which increases the PSU molecular chains’ mobility. While the stable behavior of the storage modulus of the composites heated at 350 °C up to a glass transition temperature of the PSU was shown, this was associated with the removal of residual solvent.
- The composites dried at 110 °C showed tensile strength values as high as 3200 MPa, 3850 MPa, and 4020 MPa for 20, 30, and 40 wt.% PSU solution impregnated carbon fiber yarns, respectively. An increase in the porosity of the composites after heating to 350 °C decreased the mechanical properties. The tensile strength of the composites was 3070, 3530, and 3700 MPa for 20, 30, and 40 wt.% PSU solution impregnated composites, respectively.
- It was shown that, despite the insufficient adhesion, the produced PSU solution impregnated CF yarns showed relatively high mechanical properties, realizing up to 80% of the carbon fibers tensile strength, which can be attributed to good wettability and uniform polymer matrix distribution throughout the entire volume of the composites.

## Figures and Tables

**Table 1:** Technical data of the used carbon yarns from the manufacturers.

	Number of Filaments [-]	Linear Density of the Yarn [tex]	Density [g/cm^3^]	Filament Diameter [µm]	Tensile Strength [MPa]	Tensile Modulus [GPa]	Sizing Type
Toray T700SK	12,000	800	1.8	7	4900	230	Epoxy

**Table 2:** Fiber mass fractions and theoretical densities of the PSU impregnated CF yarns.

PSU Solution	Fiber Mass Fraction, %		Theoretical Density, g/cm^3^	
	Drying	Drying + Heating	Drying	Drying + Heating
20 wt.%	71.8 ± 1.5	74.9 ± 0.9	1.592 ± 0.009	1.613 ± 0.006
30 wt.%	62.8 ± 1.2	67.0 ± 0.4	1.535 ± 0.004	1.561 ± 0.003
40 wt.%	57.7 ± 1.1	62.3 ± 1.3	1.505 ± 0.007	1.532 ± 0.007

## Data Availability

The data presented in this study are available on request from the corresponding author.
